# Increasing eccentric contraction duration enhances resistance exercise‐induced inhibitory control improvement while reducing the exertion perception: A pilot study in young men

**DOI:** 10.14814/phy2.70103

**Published:** 2024-12-11

**Authors:** Kento Dora, Takeshi Hashimoto, I. Wayan Yuuki, Su Yang, Kousei Tachi, Kaito Hashimoto, Shumpei Fujie, Motoyuki Iemitsu, Shigehiko Ogoh

**Affiliations:** ^1^ Department of Biomedical Engineering Toyo University Asaka Saitama Japan; ^2^ Faculty of Sport and Health Science Ritsumeikan University Kusatsu Shiga Japan

**Keywords:** cognitive function, eccentric contraction, exercise adherence, lactate, prefrontal cortex neural activation, resistance exercise

## Abstract

Low‐intensity resistance exercise with slow movement and tonic force generation (LRST) effectively improves cognitive inhibitory control (IC) while heightening the subjective perception, which is a barrier to exercise adherence. Compared with concentric (CON) contractions, eccentric (ECC) contractions have greater brain activation related to cognitive functions while decreasing subjective perception. Therefore, we examined whether LRST with a longer duration of ECC contraction (ECC‐LRST) could further enhance exercise‐induced IC improvement while reducing the subjective perception, compared with traditional LRST. Fourteen healthy, young males performed both ECC‐LRST and LRST, with 30% of their one‐repetition maximum. The subjective perceptions of exertion and pain associated with exercise were assessed. IC was evaluated at baseline, immediately post‐exercise, and 15‐min post‐exercise. IC improved immediately after both ECC‐LRST and LRST (both *P*s < 0.05). However, the improvement in IC persisted until 15 min post‐exercise for ECC‐LRST compared with baseline (*p* = 0.031) but not for LRST, which showed a significantly smaller improvement than ECC‐LRST (*p* = 0.042). A lower perceived pain (*p* = 0.039) and a trend toward a lower perceived exertion (*p* = 0.078) were observed during ECC‐LRST than during LRST. ECC‐LRST is an effective resistance exercise protocol for improving IC while reducing the perception of exertion.

## 1 INTRODUCTION

Habitual exercise training (i.e., aerobic exercise and resistance exercise) (Ludyga et al., 2020; Northey et al., 2018; Verburgh et al., 2014) and even acute exercise (Tsukamoto, Suga, et al., 2017; Hashimoto et al., 2018; Voss et al., 2020; Dora et al., 2021a, 2021b) are useful strategies for enhancing cognitive function in various populations. Given that muscle mass decline with aging contributes to a deterioration of quality of life (Trombetti et al., 2016) and cognitive function (Tessier et al., 2022), resistance exercise, which can increase both skeletal muscle mass (American College of Sports Medicine. American College of Sports Medicine position stand, 2009) and cognitive function (Ludyga et al., 2020; Northey et al., 2018; Verburgh et al., 2014), should be encouraged from a public health perspective. However, the mechanism behind exercise‐induced improvements in cognitive function remains unclear, and an optimal exercise protocol for enhancing cognitive function across different populations has not yet been established owing to the influence of various factors, such as exercise mode, exercise intensity, and individual conditions on outcomes (Herold et al., 2019).

Specifically, for elderly individuals, moderate‐ to high‐intensity resistance exercise (70% ~ 85% one‐repetition maximum [1‐RM]) is recommended to increase cognitive function (Northey et al., 2018), as this type of exercise also increases muscle mass and counteracts sarcopenia (American College of Sports Medicine. American College of Sports Medicine position stand, 2009). Indeed, our study confirmed that exercise‐induced improvement in cognitive function is greater at higher intensities than at lower intensities in localized resistance exercise (Tsukamoto, Suga, et al., 2017). However, moderate‐ to high‐intensity resistance exercise (>70% [1‐RM]) decreases vascular function (Miyachi, 2013) and is not recommended for populations with joint and bone disorders because of the risk of adverse events (Beck et al., 2017; Messier et al., 2021). To find an adequate exercise method to reduce these issues, we focused on low‐intensity resistance (LRE) exercise with increased muscle contraction durations (i.e., LRE exercise with slow movement and tonic force generation consisting of equal concentric contractions [i.e., 3 s] and eccentric contractions [i.e., 3 s]; LRST) (Dora et al., 2021a, 2021b), as it has been demonstrated that the LRST improves rather than decreases vascular function (Okamoto et al., 2009). The LRST has been shown to increase muscle size and strength to a degree comparable to that of high‐intensity resistance exercise (Tanimoto et al., 2008; Tanimoto & Ishii, 2006), unlike LRE at normal speed (Tanimoto et al., 2008; Tanimoto & Ishii, 2006; Watanabe et al., 2014). We have recently demonstrated that LRST is more effective than LRE for improving inhibitory control (IC), an integral component of executive function, a higher‐order cognitive function (Diamond, 2013) and achieves effects comparable to those of high‐intensity resistance exercise (Dora et al., 2021a, 2021b). Therefore, LRST might be a superior resistance exercise method that provides benefits for both skeletal muscle and cognitive function comparable to high‐intensity resistance exercise while also improving vascular function. However, one potential concern is that the subjective perception of effort and perceived leg pain are significantly greater in the LRST than in the LRE (Dora et al., 2021b) and are comparable to those experienced with high‐intensity resistance exercise (Dora et al., 2021a). Since increased subjective exertion and pain are known barriers to exercise adherence (Hurley et al., 2018; Jack et al., 2010; Trost et al., 2002), it is necessary to establish resistance exercise protocols that offer equal or greater exercise effectiveness than LRST but with a lower subjective perception of effort.

In this context, exploring a new exercise modality may be valuable. Previous studies have demonstrated that the RPE and perceived pain values are lower during eccentric (ECC) contractions than during concentric (CON) contractions at the same absolute load (Hollander et al., 2003; Miller et al., 2009). Additionally, a recent review indicated that the most favorable approach for muscle hypertrophy is a combination of slower movement during the ECC phase with faster movement during the CON phase (Wilk et al., 2021). Therefore, compared with LRST with equal durations for the ECC and CON phases, LRST with longer ECC contractions and shorter CON contractions might have a greater effect on skeletal muscle mass with less subjective effort perception during exercise. Moreover, post‐exercise prefrontal cortex activity is thought to support the effects of exercise‐induced IC improvement (Byun et al., 2014; Yanagisawa et al., 2010), and prefrontal cortex activation has been shown to be greater during ECC contractions than during CON contractions (Borot et al., 2024; Kwon & Park, 2011). These findings suggest that, compared with the traditional LRST with equal contraction durations, LRST with longer ECC contractions and shorter CON contractions might be more effective at improving cognitive function and skeletal muscle mass.

Given this background, we hypothesized that modifying the LRST modality to incorporate longer ECC contractions into shorter CON contractions (ECC‐LRST) would further advance exercise‐induced IC improvement due to greater prefrontal cortex activation. To test this hypothesis, we compared the effects of ECC‐LRST and LRST on the acute improvement of IC. IC is an aspect of goal‐directed behavior that denotes the ability to suppress internal and external distractions to successfully attend to a task at hand (Miyake et al., 2000), and young adults with better IC report higher life satisfaction and lower depression (Lee & Chao, 2012). Thus, in the present study, we focused on the IC of cognitive function.

## 2 METHODS

### 2.1 Participants

Fourteen healthy young men (age: 22 ± 2 years, body height: 175 ± 7 cm, body weight: 66 ± 10 kg, one‐repetition maximum [1‐RM] of bilateral knee extension: 119.0 ± 24.5 kg) were informed of the experimental procedures and potential risks and provided written informed consent to participate in the study. Prior to this study, we calculated the required sample size utilizing an effect size of 0.42, an α‐level of 0.01, and a β‐level of 0.2 (80% power) on the basis of the data (i.e., the reverse‐Stroop interference score) of our previous study (Dora et al., 2021b). The calculated necessary number of subjects was 14; therefore, the number of subjects recruited in this study was sufficient for ensuring statistical power and sensitivity. No participants had any known neurologic, cardiovascular, or pulmonary disorders; color blindness; or abnormal vision. The participants were instructed to avoid strenuous physical activity and abstain from caffeine and alcohol for 24 h before each experimental treatment. Moreover, the participants abstained from food for 12 h (overnight fasting) before each experiment and were not taking any medications that would affect cognitive function. This study was conducted in accordance with the guidelines of the Declaration of Helsinki. All procedures were approved by the Ethics Committee of Ritsumeikan University (BKC‐LSMH‐2023‐116).

### 2.2 Experimental procedure

Before the day of the experiment, all the participants practiced color‐word Stroop task (CWST)‐measured IC until they achieved consistent scores. The subjects subsequently underwent 1RM measurement of bilateral knee extension. Afterward, they underwent experiments for 2 days (i.e., treatments) with a wash‐out period of at least 3 days.

**FIGURE 1:**
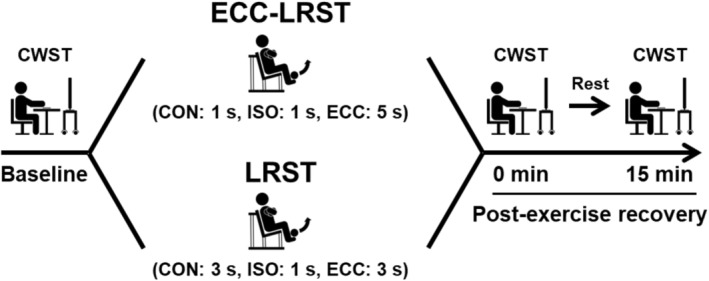
Overview of the experimental protocol.

On the experimental days (Figure 1), upon participant arrival, the participants also practiced the CWST again to minimize the learning effect. After the patients rested in the supine position for at least 10 min, their baseline data were collected. Next, the participants completed either the LRST or the ECC‐LRST in a randomized and counterbalanced manner. The cardiovascular and perceived exertion parameters during exercise sessions were measured in every set. The CWST was performed again immediately after the completion of the exercise session and at a 15‐min post‐exercise recovery period to evaluate the sustainable effects of post‐exercise IC improvements. Blood samples used to measure blood glucose and lactate levels were collected immediately after all CWSTs. Scores on the felt arousal scale (FAS) and visual analog scale (VAS) were determined immediately after all CWSTs were completed to assess the effects of psychological conditions on IC.

### 2.3 Exercise protocols

The ECC‐LRST and LRST were set at 30% 1‐RM, on the basis of previous studies (Dora et al., 2021b; Watanabe et al., 2014). Both protocols were programmed with bilateral knee extension for six sets with 10 repetitions per set using a leg extension machine (Life Fitness; Schiller Park, IL, USA). The ECC‐LRST consists of longer ECC contractions (i.e., 5 s), shorter CON contractions (i.e., 1 s) and isometric contractions [ISO] (i.e., 1 s), and the LRST consists of CON and ECC contractions of equal duration (i.e., 3 s) and ISO actions (i.e., 1 s) (Dora et al., 2021a, 2021b; Goto et al., 2009). The rest intervals between sets for both protocols lasted 1 min.

## 3 MEASUREMENTS

### 3.1 One‐repetition maximum

The subject's 1‐RM was determined by the successful lifting of the bilateral knee extension exercise on the familiarization visit day, as in our previous study (Tomoo et al., 2020, 2021; Dora et al., 2021a, 2021b). The 1‐RM value was used to determine the loads for the ECC‐LRST and LRST treatments. The 1‐RM trial was designed using increments of 10 kg until 60%–80% of the perceived maximum was achieved. The load was then gradually increased by 1–5 kg until lift failure, at which point the subject was not able to maintain the proper form or to completely lift the weight. The last acceptable lift with the highest possible load was defined as the 1‐RM. The mean load of the 30% 1‐RM for both ECC‐LRST and the LRST was 35.7 ± 7.3 kg.

### 3.2 Inhibitory control

The CWST was administered to determine the IC, as previously described (Dora et al., 2021a, 2021b). In brief, for each task, 24 stimulus words that consisted of four color names (red, blue, green, and yellow, in Japanese characters) were randomly presented on a display. All participants repeatedly performed each of the three types of CWSTs three times (i.e., nine times per test); the CWST types included congruent, neutral, and incongruent tasks (e.g., the word red presented in red, black, and blue/yellow/green font, respectively). The participants were instructed to press the color‐labeled key that corresponded to the text meaning. The total reaction times (RT) of all 24 stimulus words and response accuracy (RA) were collected for analysis. EF was assessed via the reverse‐Stroop interference score, which is defined as the difference between the average RTs on the neutral and incongruent tasks (Dora et al., 2021a, 2021b). The reverse‐Stroop interference score was calculated as [(RT on the incongruent task—RT on the neutral task)/RT on the neutral task × 100] (Ikeda et al., 2010).

### 3.3 Blood metabolites

Blood glucose and lactate levels were measured using a glucose analyzer (GlucoCard PRIME; Arkray, Kyoto, Japan) and a lactate analyzer (Lactate Pro 2; Arkray, Kyoto, Japan), respectively.

### 3.4 Cardiorespiratory variables

Beat‐to‐beat arterial blood pressure was monitored continuously via finger photoplethysmography (Finometer Pro; Finapres Medical Systems, Amsterdam, Netherlands), with a cuff placed on the middle finger of the right hand. Heart rate (HR) was calculated from each pulse wave interval. During the exercise session, the mean arterial pressure (MAP), systolic blood pressure (SBP), diastolic blood pressure (DBP), and HR were collected immediately after each set, and the mean values of all six sets were calculated for analysis.

### 3.5 Psychological conditions

Borg's rating of perceived exertion (RPE) scale, which ranges from 6 (no exertion) to 20 (maximal exertion), was used to assess the perceived exertion expended during exercise (Borg, 1982). The Borg category ratio scale (CR‐10), which ranges from 0 (nothing at all) to 10 (very, very strong), was also used to assess leg pain expended during exercise (Borg, 1982). During the exercise session, RPE and CR‐10 were collected immediately after each set, and the mean values of all six sets were calculated for analysis.

To assess the influence of arousal on cognitive function, the arousal level was measured immediately after the completion of the CWST via the FAS, which is a 6‐point, single‐item scale ranging from 1 (low arousal) to 6 (high arousal) (Svebak & Murgatroyd, 1985). Similarly, a VAS was used to assess each of the following psychological conditions: mental fatigue, ability to concentrate, and motivation. Each VAS was labeled from 0 (not at all) to 100 mm (extremely), and the participants drew lines on the VAS to indicate their psychological state during the cognitive tests (Dora et al., 2021a, 2021b).

### 3.6 Statistical analysis

All the data are expressed as the means ± SDs if a normal data distribution was confirmed via Shapiro–Wilk tests. Nonnormally distributed data are expressed as the median (IQR). The data were analyzed via two‐way (treatment × time) repeated‐measures analysis of variance (ANOVA) after a normal data distribution was confirmed. If the sphericity assumption was not met, Greenhouse–Geisser corrections were used. Partial eta‐squared ($\eta_{p}^{2}$) values were determined as a measure of the effect size for the main effect. In addition, specific differences between time points were identified via paired *t*‐tests with Benjamini–Hochberg correction. If normality was not confirmed, after confirming that there were no differences in the baseline data between treatments, the time effect for each treatment was checked for changes via one‐way repeated‐measures ANOVA or the Friedman test. If one‐way ANOVA or the Friedman test revealed a significant main effect, specific differences between time points were identified by paired *t*‐tests or Wilcoxon signed rank tests, and the *p*‐values were adjusted via Benjamini–Hochberg correction for multiple comparisons. Additionally, if a one‐way repeated‐measures ANOVA or Friedman test confirmed a main effect, the absolute change in these variables from baseline to post‐exercise (post‐EX 0–baseline, post‐EX 15–baseline) was compared between ECC‐LRST and LRST via paired *t*‐tests or Wilcoxon signed rank tests to examine differences in the exercise effects of the two treatments. The mean values of cardiovascular (i.e., HR and blood pressure) and psychological parameters (i.e., RPE and CR‐10) during exercise were compared between the ECC‐LRST treatment and the LRST treatment via paired *t*‐tests or Wilcoxon signed rank tests. Since there were missing values in the cardiovascular data, the mean value was calculated using only the values that could be obtained. The statistical significance level was defined at *p* < 0.05. For normal data distribution, Cohen's *d* effect size using the means and pooled SD were calculated, along with the 95% confidence interval to determine the magnitude of differences. The strength of the effect size of Cohen's *d* was interpreted as weak (0.20 ≤ *d* < 0.50), medium (0.50 ≤ *d* < 0.80), and large (0.80 ≤ *d*) (Cohen, 1992). For nonnormal data distribution, the effect size, as *r*, was estimated using the z‐score for the Wilcoxon signed‐rank test. The strength of the effect size of *r* was interpreted as weak (0.10 ≤ *r* < 0.30), medium (0.30 ≤ *r* < 0.50), and large (0.50 ≤ *r*) (0.50 ≤ *r*) (Cohen, 1992). All the statistical analyses were conducted via IBM SPSS software (Ver. 29.0, IBM Corp., NY, USA). In terms of the figures, individual, box‐and‐whisker, and raincloud plots were created via JASP software (version 0.18.1.0, University of Amsterdam, Netherlands).

## 4 RESULTS

Normality was confirmed at all time points only for concentration and glucose. For parameters for which normality was not confirmed, no significant differences were found between treatments at baseline (Table 1).

**TABLE 1:** Baseline states.

	Treatment		*p*‐values
	ECC‐LRST	LRST	ECC‐LRST versus LRST
Lactate (mM)	2.0 ± 0.4	1.8 ± 0.3	0.170
Color‐word Stroop tasks			
Reaction time (msec)			
Congruent task	10,687 ± 1829	10,274 ± 1217	0.300
Neutral task	11,035 ± 1569	11,471 ± 1676	0.197
Incongruent task	11,943 ± 1774	12,043 ± 1550	0.787
Response accuracy (%)			
Congruent task	97 (93–100)	99 (96–100)	0.116
Neutral task	97 (94–100)	99 (93–100)	0.607
Incongruent task	97 ± 2	98 ± 1	0.787
Interference scores (%)	8.3 ± 5.2	5.5 ± 7.7	0.634
Psychological states			
Felt arousal scale, 1–6			
Arousal	3 (2–4)	3 (1–4)	0.763
Visual analog scales, 0–100 mm			
Mental fatigue	21 (4–78)	33 (0–76)	0.345
Motivation	71 ± 23	68 ± 22	0.490

*Note*: Values are mean ± SD or median (IQR). The *p*‐values shown in the table represent the results of the comparison between ECC‐LRST and LRST treatment in baseline states by paired *t*‐test or Wilcoxon signed rank test.

### 4.1 Psychological response to exercise

A lower CR‐10 score (*p* = 0.039, *d* = 0.526; Table 2) and a trend toward a lower RPE (*p* = 0.078, *d* = 0.601) were observed during ECC‐LRST than during LRST.

**TABLE 2:** Psychological and cardiovascular responses to exercise.

	Treatment		*p*‐values
	ECC‐LRST	LRST	ECC‐LRST versus LRST
Psychological			
RPE, 6–20	13 ± 2	14 ± 2	0.078
CR‐10, 0–10	5 ± 2	6 ± 1	**0.039**
Cardiovascular			
HR	97 ± 14	94 ± 11	0.169
MAP	112 ± 11	116 ± 16	0.319
SBP	167 ± 23	164 ± 21	0.599
DBP	91 ± 9	94 ± 17	0.277

*Note*: Values are mean ± SD. The *p*‐values shown in the table represent the results of the comparison between ECC‐LRST and LRST treatment at exercise by paired *t*‐test. Significant values are in bold.

### 4.2 Cardiovascular response to exercise

The cardiovascular response (*p* = 0.169, *d* = 0.280 for HR; *p* = 0.319, *d* = 0.232 for MAP; *p* = 0.599, *d* = 0.149 for SBP; *p* = 0.277, *d* = 0.266 for DBP; Table 2) did not differ between treatments.

### 4.3 Changes in blood metabolites

Glucose data were analyzed via two‐way (Treatment × Time) repeated‐measures ANOVA because a normal data distribution was confirmed. There was a significant main effect of time (*p* =  0.011, $\eta_{p}^{2}$ = 0.292; Table 3) but not a significant main effect of treatment (*p* = 0.610, $\eta_{p}^{2}$ = 0.021) or a significant treatment × time interaction (*p* = 0.270, $\eta_{p}^{2}$ = 0.096). Compared with that at baseline, the glucose level decreased immediately after exercise in both treatments (*p* = 0.022, *d* = 2.218 at post‐EX 0; *p* = 0.667, *d* = 0.351 at post‐EX 15).

**TABLE 3:** Blood metabolites, cognitive tasks and psychological conditions throughout ECC‐LRST, and LRST treatments.

	Time points			*p*‐values			
	Baseline	Post‐EX 0	Post‐EX 15	One‐way ANOVA or Friedman test	Two‐way ANOVA		
					Treatment	Time	Interaction
Blood metabolites							
Glucose (mg/dL)							
ECC‐LRST	91 ± 7	86 ± 6^†^	92 ± 7^‡^	N/A	0.610	**0.011**	0.270
LRST	89 ± 5	88 ± 5^†^	89 ± 8^‡^	N/A			
Lactate (mM)							
ECC‐LRST	1.9 (1.4–3.0)	3.3 (2.2–9.5)^†^	2.3 (1.2–7.3)^†^ ^,^ ^‡‡^	**<0.001**	N/A	N/A	N/A
LRST	1.9 (1.2–2.6)	3.4 (2.4–9.5)^††^	2.5 (1.6–7.3)^††^ ^,^ ^‡‡^	**<0.001**			
Color‐word Stroop tasks							
Reaction time (msec)							
Congruent task							
ECC‐LRST	10,687 ± 1829	9898 ± 1614^†^	10,349 ± 1914	**0.049**	N/A	N/A	N/A
LRST	10,223 (8055–14,581)	9396 (7962–13,757)	9897 (8102–14,961)	0.931			
Neutral task							
ECC‐LRST	10,890 (8837–14,316)	10,104 (8660–15,439)	10,363 (8673–15,024)	0.395	N/A	N/A	N/A
LRST	11,657 (8781–14,702)	10,535 (8540–18,321)	10,647 (8685–17,878)	0.071			
Incongruent task							
ECC‐LRST	11,534 (9756–15,483)	10,353 (8365–14,530)^††^	10,577 (9105–15,766)^††^	**0.002**	N/A	N/A	N/A
LRST	11,856 (9434–15,224)	10,329 (8995–13,909)^†^	11,537 (9100–20,141)	**0.008**			
Response accuracy (%)							
Congruent task							
ECC‐LRST	97 (93–100)	99 (92–100)	97 (94–99)	0.646	N/A	N/A	N/A
LRST	99 (96–100)	97 (93–100)	97 (92–100)^†^	**0.025**			
Neutral task							
ECC‐LRST	97 (94–100)	98 (94–100)	99 (96–100)	0.754	N/A	N/A	N/A
LRST	99 (93–100)	98 (93–100)	99 (94–100)	0.587			
Incongruent task							
ECC‐LRST	98 (92–100)	99 (94–100)	99 (96–100)	0.558	N/A	N/A	N/A
LRST	99 (96–100)	99 (90–100)	97 (93–100)	0.171			
Psychological states							
Felt arousal scale, 1–6							
Arousal (N/A)							
ECC‐LRST	3 (2–4)	4 (2–5)^††^	4 (2–5)^††^	**<0.001**	N/A	N/A	N/A
LRST	3 (1–4)	4 (2–5)^††^	4 (2–5)^††^	**<0.001**			
Visual analog scales, 0–100 mm							
Mental fatigue							
ECC‐LRST	21 (4–78)	44 (5–89)^††^	48 (16–93)^††^	**<0.001**	N/A	N/A	N/A
LRST	33 ± 24	40 ± 26	39 ± 26	0.207			
Concentrate							
ECC‐LRST	69 ± 22	72 ± 24	64 ± 27	N/A	0.202	0.489	0.277
LRST	60 ± 22	66 ± 25	65 ± 21	N/A			
Motivation							
ECC‐LRST	73 (18–100)	82 (5–100)	69 (2–100)	0.487	N/A	N/A	N/A
LRST	68 ± 22	68 ± 22	66 ± 24	0.807			

*Note*: Values are mean ± SD or median (IQR). The *p*‐values shown in the table represent the results of two‐way analysis of variance, one‐way analysis of time or Friedman test, and the letters † and ‡ represent the results of the comparison between time points by paired *t*‐test or Wilcoxon signed rank test with Benjamini‐Hochberg correction. Significant values are in bold. post‐EX 0; immediately after exercise; post‐EX 15; 15‐min at post‐exercise recovery period.

^†^ *p* < 0.05 versus baseline.

^††^ *p* < 0.01 versus baseline.

^‡^ *p* < 0.05 versus post‐EX 0.

^‡‡^ *p* < 0.01 versus post‐EX 0.

For both treatments, there was a significant main effect of time on lactate (*p* < 0.001 for ECC‐LRST; *p* < 0.001 for LRST; Table 3). The lactate level increased both immediately after exercise and 15 min post‐exercise compared with baseline in the two treatments (*p* = 0.003, *r* = 0.881 at post‐EX 0 for ECC‐LRST; *p* = 0.025, *r* = 0.598 at post‐EX 15 for ECC‐LRST; *p* = 0.003, *r* = 0.881 at post‐EX 0 for LRST; *p* = 0.006, *r* = 0.740 at post‐EX 15 for LRST), indicating that blood levels of lactate increased after both exercise treatments. The extent of changes from baseline to after exercise did not differ between treatments (*p* = 0.106, *r* = 0.432 at post‐EX 0–baseline; *p* = 0.130, *r* = 0.405 for post‐EX 15–baseline; Table 4).

**TABLE 4:** Comparison of changes in lactate, cognitive tasks, and psychological conditions between ECC‐LRST and LRST treatment.

	Treatment		*p*‐values
	ECC‐LRST	LRST	ECC‐LRST versus LRST
Lactate (mM)			
Δpost‐EX 0–baseline	1.5 (0.2–7)	1.7 (0.4–6.9)	0.106
Δpost‐EX 15–baseline	0.6 (−0.6–4.8)	0.6 (−0.4–4.7)	0.130
Color‐word Stroop tasks			
Reaction time (msec)			
Congruent task			
Δpost‐EX 0–baseline	−1076 (−2312–1788)	−19 (−2260–6575)	0.158
Δpost‐EX 15–baseline	−338 ± 1104	−6 ± 1127	0.253
Incongruent task			
Δpost‐EX 0–baseline	−1004 ± 702	−1027 ± 971	0.944
Δpost‐EX 15–baseline	−805 (−2533–283)	−590 (−2956–6563)	0.140
Response accuracy (%)			
Congruent task			
Δpost‐EX 0–baseline	0 ± 3	−1 ± 2	0.231
Δpost‐EX 15–baseline	0 ± 3	−2 ± 3	**0.033**
Psychological states			
Felt arousal scale, 1–6			
Arousal (N/A)			
Δpost‐EX 0–baseline	1 (0–2)	1 (−1–3)	0.454
Δpost‐EX 15–baseline	1 (0–2)	1 (0–3)	0.206
Visual analog scales, 0–100 mm			
Mental fatigue			
Δpost‐EX 0–baseline	16 ± 12	7 ± 15	0.147
Δpost‐EX 15–baseline	26 ± 17	7 ± 21	**0.011**

*Note*: Values are mean ± SD or median (IQR). The *p*‐values shown in the table represent the results of the comparison of changes from baseline to each time point (∆) between ECC‐LRST and LRST treatment in cognitive tasks and psychological condition by paired *t*‐test or Wilcoxon signed rank test. Significant values are in bold.

### 4.4 Changes in color‐word Stroop task (CWST)‐measured IC

For ECC‐LRST treatment, there was a significant main effect of time on the RT in the congruent task (*p* = 0.049, $\eta_{p}^{2}$ = 0.206; Table 3) but not in the LRST treatment (*p* = 0.931). The RT in the congruent task was shorter immediately after ECC‐LRST than at baseline (*p* = 0.031, *d* = 0.458), but the extent of changes from baseline to immediately after exercise did not differ between treatments (*p* = 0.158, *r* = 0.377 at post‐EX 0–baseline; *p* = 0.253, *d* = 0.298 at post‐EX 15–baseline; Table 4). For the LRST treatment, there was a significant main effect of time on RA in the congruent task (*p* = 0.025) but not for the ECC‐LRST treatment (*p* = 0.646). Compared with that at baseline, the RA in the congruent task decreased at 15 min after the LRST (*p* = 0.036, *r* = 0.673), and the extent of the decrease from baseline to 15 min after exercise was greater after the LRST than after ECC‐LRST (*p* = 0.033, *d* = 0.796). There was no significant main effect of time on the RT (*p* = 0.395 for the ECC‐LRST; *p* = 0.071 for the LRST) or RA (*p* = 0.754 for the ECC‐LRST; *p* = 0.587 for the LRST) in the neutral task for either treatment. For both treatments, there was a significant main effect of time on the RT in incongruent tasks (*p* = 0.002 for the ECC‐LRST; *p* = 0.008 for the LRST). Compared with that at baseline, the RT in the incongruent task in the ECC‐LRST treatment was shorter both immediately after exercise and 15 min post‐exercise (*p* = 0.003, *r* = 0.830 at post‐EX 0; *p* = 0.003, *r* = 0.864 at post‐EX 15), whereas in the LRST treatment, the RT was shorter immediately after exercise than at baseline but returned to baseline at 15 min after exercise (*p* = 0.012, *r* = 0.780 at post‐EX 0; *p* = 0.053, *r* = 0.562 at post‐EX 15). The extent of changes in the RT from baseline to immediately after exercise in the incongruent task did not differ between treatments (*p* = 0.944, *d* = 0.027 at post‐EX 0–baseline; *p* = 0.140, *r* = 0.394 at post‐EX 15–baseline). For both treatments, there was no significant main effect of time on the RA in the incongruent task (*p* = 0.558 for ECC‐LRST; *p* = 0.171 for LRST).

**FIGURE 2:**
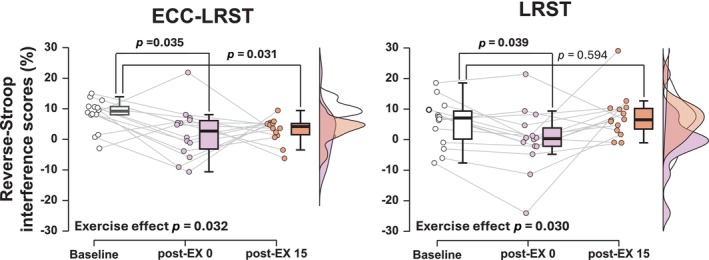
Reverse‐Stroop interference scores throughout the ECC‐LRST and LRST treatments. Left: Changes in reverse‐Stroop interference scores over time throughout ECC‐LRST. Right: The changes in reverse‐Stroop interference scores over time throughout LRST. The raincloud plots show the distribution of the reverse‐Stroop interference score, the circle plots represent individual data, and the box‐and‐whisker plots represent the median values (IQR and max/min).

**FIGURE 3:**
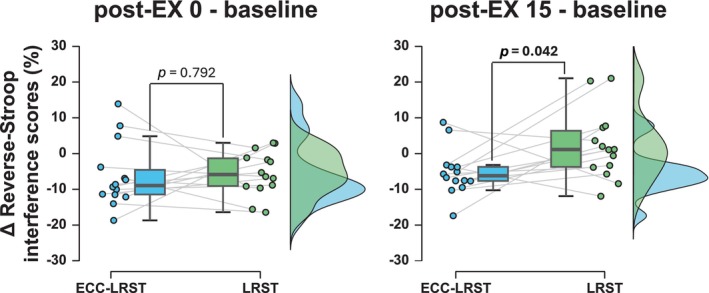
Comparison of changes in the reverse‐Stroop interference scores between the ECC‐LRST and LRST treatments. Comparison of changes from baseline to each time point (∆) between ECC‐LRST and LRST treatments in terms of reverse‐Stroop interference scores. Left: Changes in the reverse‐Stroop interference score from baseline to immediately after exercise (i.e., post‐EX 0—baseline). Right: Changes in the reverse‐Stroop interference score from baseline to 15 min after exercise (i.e., post‐EX 15—baseline). Blue indicates participants who performed ECC‐LRST, and green indicates participants who performed LRST. The raincloud plots show the distribution of the ∆reverse‐Stroop interference score, the circle plots represent individual data, and the box‐and‐whisker plots represent the median values (IQR and max/min).

For both treatments, there was a significant main effect of the reverse‐Stroop interference score (*p* = 0.032, $\eta_{p}^{2}$= 0.232 for ECC‐LRST; *p* = 0.030 for LRST; Figure 2). In the ECC‐LRST treatment, the reverse‐Stroop interference score was lower both immediately after exercise and 15 min post‐exercise than at baseline (*p* = 0.035, *d* = 0.899 at post‐EX 0; *p* = 0.031, *d* = 1.130 at post‐EX 15), whereas in the LRST treatment, the reverse‐Stroop interference score was lower immediately after exercise than at baseline but returned to baseline at 15 min after exercise (*p* = 0.039, *r* = 0.663 at post‐EX 0; *p* = 0.594, *r* = 0.143 at post‐EX 15). This analysis revealed that IC significantly improved immediately after both ECC‐LRST and LRST compared with that before each exercise, and the improvement remained significant until 15 min after ECC‐LRST but not after LRST. The extent of changes in the reverse‐Stroop interference score from baseline to 15 min after exercise was greater after ECC‐LRST than after LRST (*p* = 0.042, *d* = 0.903; Figure 3), although there was no difference immediately after exercise (*p* = 0.792, *d* = 0.103).

### 4.5 Changes in psychological conditions for cognitive tasks

There was a significant main effect of time on arousal (*p* < 0.001 for the ECC‐LRST; *p* < 0.001 for the LRST; Table 3) for both treatments. Arousal was greater both immediately after exercise and 15 min post‐exercise than at baseline in the two treatments (*p* = 0.003, *r* = 0.878 at post‐EX 0 for ECC‐LRST; *p* = 0.005, *r* = 0.806 at post‐EX 15 for ECC‐LRST; *p* = 0.009, *r* = 0.791 at post‐EX 0 for LRST; *p* = 0.003, *r* = 0.838 at post‐EX 15 for LRST), and the extent of the change from baseline to after exercise did not differ between treatments (*p* = 0.454, *r* = 0.200 at post‐EX 0–baseline; *p* = 0.206, *r* = 0.338 at post‐EX 15–baseline; Table 4). For ECC‐LRST, there was a significant main effect of mental fatigue (*p* < 0.001), but not for LRST (*p* = 0.207, $\eta_{p}^{2}$ = 0.118). Compared with that at baseline, mental fatigue was greater both immediately after exercise and 15 min post‐exercise for ECC‐LRST‐treated participants (*p* = 0.003, *r* = 0.850 at post‐EX 0; *p* = 0.002, *r* = 0.881 at post‐EX 15), and the extent of changes in mental fatigue from baseline to 15 min after exercise was greater after ECC‐LRST treatment than after LRST treatment (*p* = 0.011, *d* = 0.995). The concentration data were analyzed via two‐way (Treatment × Time) repeated‐measures ANOVA because a normal data distribution was confirmed. No significant main effects of time (*p* = 0.489, $\eta_{p}^{2}$ = 0.054), treatment (*p* = 0.202, $\eta_{p}^{2}$ = 0.122), or the treatment × time interaction (*p* = 0.277, $\eta_{p}^{2}$ = 0.094) were found for concentration. There was no significant main effect of time on motivation for either treatment (*p* = 0.487 for ECC‐LRST; *p* = 0.807, $\eta_{p}^{2}$ = 0.010 for LRST).

## 5 DISCUSSION

The primary finding of this study was that, although IC (i.e., the reverse‐Stroop interference score) improved immediately after both ECC‐LRST and LRST compared with baseline, the improvement remained significant until 15 min after ECC‐LRST but not after LRST. Furthermore, the degree of improvement in IC at 15 min post‐exercise was significantly greater after ECC‐LRST than after LRST. Additionally, perceived pain and exertion levels were lower during ECC‐LRST than during LRST. These findings suggest that, compared with LRST, ECC‐LRST is a more effective protocol for improving IC while maintaining lower levels of perceived effort. This is particularly noteworthy, as LRST itself is already recognized as a superior resistance exercise protocol because of its positive effects on skeletal muscle mass (Tanimoto et al., 2008; Tanimoto & Ishii, 2006; Watanabe et al., 2014), vascular function (Okamoto et al., 2009), and cognitive function (Dora et al., 2021a, 2021b).

We found that ECC‐LRST exerted beneficial effects on cognitive function, thus supporting our hypothesis. We observed a significant improvement in the immediate post‐exercise IC (i.e., immediate effect) compared with baseline levels in both the ECC‐LRST and LRST treatments. However, the improvement in IC lasted for up to 15 min post‐exercise after ECC‐LRST but not after LRST, indicating that resistance exercise—particularly ECC contractions—is effective for improving IC. In addition to the immediate effect, the sustainable effect (i.e., duration of significant improvements in post‐exercise IC) is an important factor for understanding the potential impact of various resistance exercise protocols on post‐exercise IC improvements (Dora et al., 2021a, 2021b; Tomoo et al., 2020, 2021). Previous studies that examined the same population as the present study found that exercise‐induced improvement in IC was sustained for up to 20–30 min after both high‐volume LRE exercise and volume‐matched high‐intensity resistance exercise, with large effect sizes (all *d*s = 0.80 to 1.40 for post‐EX 0 to post‐EX 30 vs. baseline in both exercise protocols) (Dora et al., 2021a; Tomoo et al., 2020); similar findings were reported after ECC‐LRST (*d* = 0.899 for post‐EX 0 vs. baseline; *d* = 1.130 at post‐EX15 vs. baseline). Thus, despite the low volume and low intensity of exercise, ECC‐LRST may yield post‐exercise improvement in IC comparable to that of high‐volume or high‐intensity resistance exercise protocols. Additionally, previous studies reported that when exercise volume is comparable, there is no significant difference in the degree of cognitive function improvement immediately after exercise, regardless of exercise intensity (Tomoo et al., 2020; Tsukamoto et al., 2016). These findings suggest that exercise volume, rather than intensity, may be a more important factor in the degree of IC improvement immediately after exercise. On the other hand, in a previous study focusing on muscle contraction modality, we compared the effects of resistance exercise with slow velocity muscle contraction and normal velocity muscle contraction on post‐exercise IC with a matched exercise volume (Dora et al., 2021b). We found similar improvements in IC immediately after both muscle contraction velocities compared with before exercise; however, the improvement was sustained until 20 min after resistance exercise with slow velocity muscle contraction but not after normal velocity muscle contraction. Considering these previous findings, in the present study, it is possible that there was no difference in IC improvement immediately after exercise between treatments because of the matched exercise volume, whereas further improvement in IC during post‐exercise recovery was achieved by modifying muscle contraction modality, such as muscle contraction type (i.e., ECC contraction). To our knowledge, only one study has examined the effects of different muscle contraction types on cognitive function (Kan et al., 2019). This study evaluated the effects of cycling exercise, primarily using ECC or CON contractions, on post‐exercise attentional function. The results suggested that there was no difference in the effects of exercise on attentional function between ECC and CON contractions. On the other hand, the present study combined CON and ECC contractions and revealed that exercise with a longer duration of ECC contraction enhanced the effect of exercise‐induced cognitive function. These results suggest that the cognitive‐enhancing effects of ECC contractions may not be due to ECC contractions alone but rather to a combination of ECC and CON contractions. Therefore, changing the modality of skeletal muscle contraction may further advance the significant cognitive improvement induced by LRST, and resistance exercise centered on ECC contraction may be particularly effective in this regard. Additionally, in addition to IC, ECC contractions may be beneficial for information processing speed, which is important in daily life functions (e.g., driving cars) (Roenker et al., 2003). The RT of the congruent task, which is an indicator of information processing ability, was shorter immediately after exercise than before exercise in the ECC‐LRST treatment but not in the LRST treatment. Although the degree of change from baseline to immediate post‐exercise varied between treatments (ECC‐LRST [−1076] vs. the LRST [−19]), no significant differences were detected, probably due to large individual differences. Since this study was designed (e.g., sample size) with IC as the primary outcome, identifying the clear effects of ECC contraction on improved information processing speed was not easy. Future studies should examine the effects of ECC contraction on various cognitive functions, including information processing, due to their importance in daily life.

The benefits of habitual exercise on cognitive function might be attributed to repeated, acute improvements in cognitive function in response to exercise (Hashimoto et al., 2021; Voss et al., 2020). Notably, the degree to which executive function is improved by acute exercise is associated with the cognitive improvement induced by chronic exercise training (Voss et al., 2020). Therefore, exploring ways to increase the extent of acute improvements in cognitive function should be useful for developing an effective regular program to improve cognitive function. In this study, both treatments improved the IC immediately after exercise, whereas only the ECC‐LRST treatment resulted in continued improvement in IC until 15 min after exercise. In this context, previous studies have shown a correlation between acute exercise‐induced cognitive improvement measured at 15 min post‐exercise and the enhancement of cognitive function by long‐term exercise training (Voss et al., 2020). Therefore, it is plausible that the ECC‐LRST may also be more effective in improving cognitive function through long‐term exercise training, but further research is needed to verify this issue.

No significant differences in cardiovascular responses, such as HR or blood pressure, during resistance exercise were identified between the treatments. However, lower subjective perceived exertion and leg pain levels were observed during the ECC‐LRST than during the LRST. In support of these results, previous studies revealed lower levels of perceived exertion and pain for ECC contraction than for CON contraction when the same absolute load was used for each contraction (Hollander et al., 2003; Miller et al., 2009), which may be attributed to the lower relative intensity of ECC contraction than CON contraction when the absolute load was used (Enoka, 1996). Similarly, it is likely that ECC‐LRST with longer ECC contraction elicits lower relative exercise intensity than LRST, which might attenuate the increase in perceived effort. The exertion perceived to be necessary for exercise is a barrier to physical activity and resistance exercise (Hurley et al., 2018; Trost et al., 2002), and perceived pain has been suggested to interfere with physical therapy adherence (Jack et al., 2010). Thus, the ECC‐LRST is not only more effective in improving cognitive function but also elicits a lower perception of exertion, making it more acceptable and suitable for long‐term intervention for various populations than the LRST.

The present study was not designed to determine the physiological mechanisms of the effects of ECC contraction on cognitive function. Therefore, the precise mechanism(s) underlying the longer ECC contraction‐enhanced cognitive improvement is unknown, but possible mechanisms are briefly discussed. Glucose and lactate are important energy sources for the human brain (Van Hall et al., 2009). Moreover, it has been suggested that lactate produced by exercise is preferentially used to meet the energy requirements of neuronal activation during exercise and is also an important substrate supporting exercise‐induced cognitive function (Hashimoto et al., 2018, 2021). In both treatments, however, similar exercise‐induced decreases in blood glucose and increases in lactate were observed. Thus, it is difficult to explain further IC improvement in the ECC‐LRST by the difference in the energy provided to the brain in the present study. On the other hand, activation of the prefrontal cortex, which has been shown to be important for improving cognitive function after exercise (Byun et al., 2014; Yanagisawa et al., 2010), may be involved in the IC results. Prefrontal cortex activation has been shown to be greater during ECC contractions than during CON contractions (Borot et al., 2024; Kan et al., 2019; Kwon & Park, 2011), although the reason for the differences in prefrontal cortex activation by contraction mode remains unclear. One possibility is that movement control is more difficult during ECC contractions than during CON contractions (Christou & Carlton, 2002) because ECC contractions have lower discharge rates of motor units than CON contractions, with selective recruitment of fewer high‐threshold motor units and derecruitment of low‐threshold motor units (Kossev & Christova, 1998; Linnamo et al., 2003). Consequently, there may be greater activation of the prefrontal cortex to movement control at higher levels, such as that encountered during ECC contractions (Perrey, 2018). Thus, ECC‐LRST may be effective in improving IC after exercise by enhancing prefrontal cortex activation due to the longer duration of ECC contraction. To further clarify this mechanism, future studies should examine whether ECC‐LRST enhances prefrontal cortex activation more than LRST, thereby contributing to further cognitive improvement.

Although an exercise‐induced increase in arousal is related to post‐exercise IC improvement (Byun et al., 2014), the arousal levels assessed by the FAS in the present study did not differ between treatments. On the other hand, only individuals in the ECC‐LRST treatment showed increased mental fatigue after exercise compared with baseline levels. Excessive mental fatigue has been suggested to impair cognitive function (Tsukamoto et al., 2022; Wang et al., 2014), but ECC‐LRST led to improved cognitive function, suggesting that the increase in mental fatigue induced by ECC‐LRST may not be excessive enough to impair cognitive function. Indeed, there were no changes in motivation or concentration on cognitive tasks in either treatment throughout the experiment, indicating that participants engaged in all CWSTs with consistent concentration and motivation irrespective of mental fatigue. On the contrary, the increase in mental fatigue induced by ECC contraction reflects the greater attentional demand required due to the difficulty of motor control, concomitant with the prefrontal cortex activation to sustain such demand (Borot et al., 2024; Kan et al., 2019), supporting our speculation that prefrontal activation is greater after ECC‐LRST than after LRST, thereby contributing to further cognitive improvement as aforementioned.

Our previous study revealed that significant improvement in post‐exercise IC compared with baseline was sustained until 20 min after LRST (Dora et al., 2021b). In contrast, in the current study, LRST yielded an immediate improvement in IC after exercise, but this improvement disappeared and IC returned to baseline levels 15 min after exercise. This inconsistency in results may be related to the different experimental conditions. The previous study included a 5‐min cycling warm‐up before resistance exercise (Dora et al., 2021b), whereas this study did not. Warm‐up exercise affects several physiological factors, such as muscle metabolism, oxygen uptake kinetics, and muscle fiber function, during subsequent exercise (McGowan et al., 2015). For example, differences in muscle temperature before exercise may affect muscle glycogen utilization during exercise (Starkie et al., 1999). Indeed, muscle metabolism in response to LRST might differ between studies as the mean change in blood lactate from pre‐exercise to immediately post‐exercise in our previous study was 4.3 mM (Dora et al., 2021b), whereas in this study, the median change was 1.7 mM. Thus, it is possible that differences in the experimental conditions of LRST (e.g., with or without warm‐up exercise) may be associated with altered effects on cognitive function, but a clear explanation is difficult. If warm‐up exercise enhances post‐exercise IC improvement, we need to consider the total exercise protocol, including warm‐up exercise, to improve IC. Further investigations are needed to explore this interesting question. However, in the present study, since neither of the experimental conditions included warm‐up exercise, this inconsistency in results does not undermine the benefits of the ECC‐LRST protocol for exercise‐induced improvement in IC.

### 5.1 Perspective

The physiological mechanisms underlying the effects of acute exercise, including resistance exercise, on cognitive function remain unclear (Huang et al., 2022). Resistance exercise differs from aerobic exercise in that it consists of multiple contraction modalities (CON contraction, ECC contraction, and ISO contraction), and furthermore, the changes in brain neural activity and metabolism that may affect cognitive function differ by contraction mode (Borot et al., 2024; Hollander et al., 2003; Kwon & Park, 2011). Indeed, the present study revealed that different contraction patterns during resistance exercise affect exercise‐induced cognitive function. Therefore, further research that compares the effects of CON contractions, ECC contractions, or a combination of the two contraction modes in resistance exercise on cognitive function and elucidates the physiological mechanisms of each contraction mode will clarify the complex physiological background of resistance exercise‐induced cognitive function improvement, thereby establishing optimal resistance exercise for improving cognitive function.

To establish efficient and effective exercise programs for improving cognitive function, most research has been conducted to clarify the effects of exercise intensity, duration, and volume on exercise‐induced improvements in cognitive function across different populations (Chang et al., 2012; Tsukamoto et al., 2016; Tsukamoto, Takenaka, et al., 2017; Tomoo et al., 2020). For example, we found that in localized resistance exercise, higher‐intensity exercise was more effective than lower‐intensity exercise in improving cognitive function (Tsukamoto, Suga, et al., 2017). On the other hand, few studies have examined the relationship between contraction mode and cognitive function improvement. Given the favorable effects of ECC contractions on exercise‐induced IC improvement as well as exercise effort shown in this study, it is reasonable to say that contraction type is also one of the important determinants of exercise effects on cognitive function and that changing contraction style is applicable to further enhancing exercise effects in various exercise modalities, including resistance exercise and aerobic exercise. In addition, whether ECC contractions are also effective in improving cognitive functions other than IC (e.g., memory function, working memory, etc.) and whether the effects of ECC contractions vary with exercise intensity, duration, volume, and different populations are also important questions.

### 5.2 Limitations

Notably, this study has several limitations. Since this study was conducted in healthy young men, the present results cannot be generalized to elderly individuals, chronically ill patients or women. Further research is needed to determine the effects of ECC‐LRST on post‐exercise IC improvement in these populations to further disseminate ECC‐LRST in clinical settings.

## 6 CONCLUSION

In the present study, compared with the LRST, ECC‐LRST resulted in less exertion and pain at the exercise site during exercise, and the post‐exercise cognitive improvement lasted longer. Therefore, ECC‐LRST is an effective resistance exercise protocol for improving IC while reducing the increased perception of effort during exercise, which can affect exercise adherence.

## AUTHOR CONTRIBUTIONS

K.D., T.H., and S.O. conceived and designed the research; K.D., I.W.Y., S.Y., K.T., and K.H. performed the experiments; K.D. collected the data; K.D. statistically analyzed the data; K.D., T.H., I.W.Y., S.Y., K.T., K.H., S.F., M.I., and S.O. interpreted the results of the experiments; K.D. prepared the figures; K.D. drafted the manuscript; and K.D., T.H., I.W.Y., S.Y., K.T., K.H., S.F., M.I., and S.O. edited and revised the manuscript. All the authors read and approved the final version of the manuscript.

## FUNDING INFORMATION

This study was funded in part by JSPS KAKENHI (grant numbers. 23K24727 to S.O.; grant numbers. 23K21635 to T.H.), and JST SPRING (grant number. JPMJSP2159 to K.D.).

## CONFLICT OF INTEREST STATEMENT

The authors declare that they have no conflict of interest.

## ETHICS STATEMENT

Subjects were fully informed about the procedures and potential risks of the experiment before giving written informed consent to participate in the study, which was approved by by the Ethics Committee of Ritsumeikan University (BKC‐LSMH‐2023‐116).

## CONSENT TO PARTICIPATE

Participants provided written informed consent before participation under the principles of the Declaration of Helsinki.

## CONSENT FOR PUBLICATION

Participants cannot be individually identified from data published in this manuscript. Participants were made aware of the intent to publish this data when providing informed consent.

## Data Availability

The datasets generated and analyzed during the current study are available from the corresponding author on reasonable request.
